# Epigastrische Schmerzen beim „Magentumor“: Der Kolibri unter den Differenzialdiagnosen

**DOI:** 10.1007/s00104-021-01522-6

**Published:** 2021-10-19

**Authors:** Ines Gockel, Wolfgang Hartmann, Hannes Köhler, Jakob Leonhardi, Simone Heyn, René Thieme

**Affiliations:** 1 Klinik und Poliklinik für Viszeral‑, Transplantations‑, Thorax- und Gefäßchirurgie, Department für Operative Medizin (DOPM), Universitätsklinikum Leipzig, AöR, Liebigstr. 20, 04103 Leipzig, Deutschland; 2 Sektion für Translationale Pathologie, Gerhard-Domagk-Institut für Pathologie, Universitätsklinikum Münster, Münster, Deutschland; 3 Innovation Center Computer Assisted Surgery (ICCAS), Universität Leipzig, Leipzig, Deutschland; 4 Klinik und Poliklinik für Diagnostische und Interventionelle Radiologie, Universitätsklinikum Leipzig, AöR, Leipzig, Deutschland; 5 Klinik und Poliklinik für Hämatologie, Zelltherapie und Hämostaseologie, Universitätsklinikum Leipzig, AöR, Leipzig, Deutschland

## Anamnese

Der 48-jährige Patient stellte sich wegen rezidivierender epigastrischer Schmerzen sowie gastroösophagealer Refluxsymptome beim niedergelassenen Gastroenterologen zur Endoskopie vor. An Vorerkrankungen waren bekannt: 1. stattgehabte *Helicobacter-pylori*(HP)-Eradikation 3 Jahre zuvor, 2. rheumatoide Arthritis (mit wöchentlicher Einnahme von 10 mg Methotrexat), 3. arterielle Hypertonie, Adipositas (BMI 36,2 kg/m^2^), 4. Diabetes mellitus Typ I („late onset“, mit ca. 40 Jahren), 5. Hyperlipidämie, 6. asymptomatische Cholezystolithiasis 6. Leistenhernienoperation als Jugendlicher, ansonsten keine weiteren Voroperationen. Es konnte ein Nikotinkonsum mit kumulativ 30 PY („pack years“) eruiert werden.

## Klinischer Befund

Bei der klinischen Untersuchung fand sich ein weiches Abdomen ohne Druckschmerz oder Resistenzen. Endoskopisch imponierte neben einer im Antrum und Korpus des Magens betonten mittelgradigen chronischen Pangastritis bei HP-Positivität ein ca. 2 × 1,2 cm durchmessender, ulzerierender Tumor. Endosonographisch bestätigte sich dessen Lokalisation im Magenkorpus (uT3, N0), computertomographisch zeigte sich der Tumor nodulär konfiguriert und kontrastmittelaffin. In der Positronenemissionstomographie(PET)-Computertomographie(CT) fanden sich keine metastasensuspekten Befunde. Nebenbefundlich stellte sich eine Porzellangallenblase mit Funduskonkrement dar. Laborchemisch wurden im Routinelabor keine pathologischen Befunde erhoben.

## Weiteres Prozedere

Histologisch ergab sich in den Biopsien der Nachweis eines teils faszikulär wachsenden spindelzelligen mesenchymalen Tumors ohne stärkergradige Kernpleomorphien. Die immunhistochemische Untersuchung ergab keinen Nachweis von CD34, CD117 oder DOG1, damit Ausschluss eines gastrointestinalen Stromatumors, kein Nachweis von Zytokeratinen, Desmin, glattmuskulärem Aktin, ERG, S‑100-Protein oder SOX10, jedoch zeigte sich eine starke Positivität für TLE‑1. In der Fluoreszenz-in-situ-Hybridisierung (FISH) gelang der Nachweis eines Rearrangements mit Beteiligung des *SS18*-Genlokus.

## Wie lautet Ihre Diagnose?

**Figure 1:**
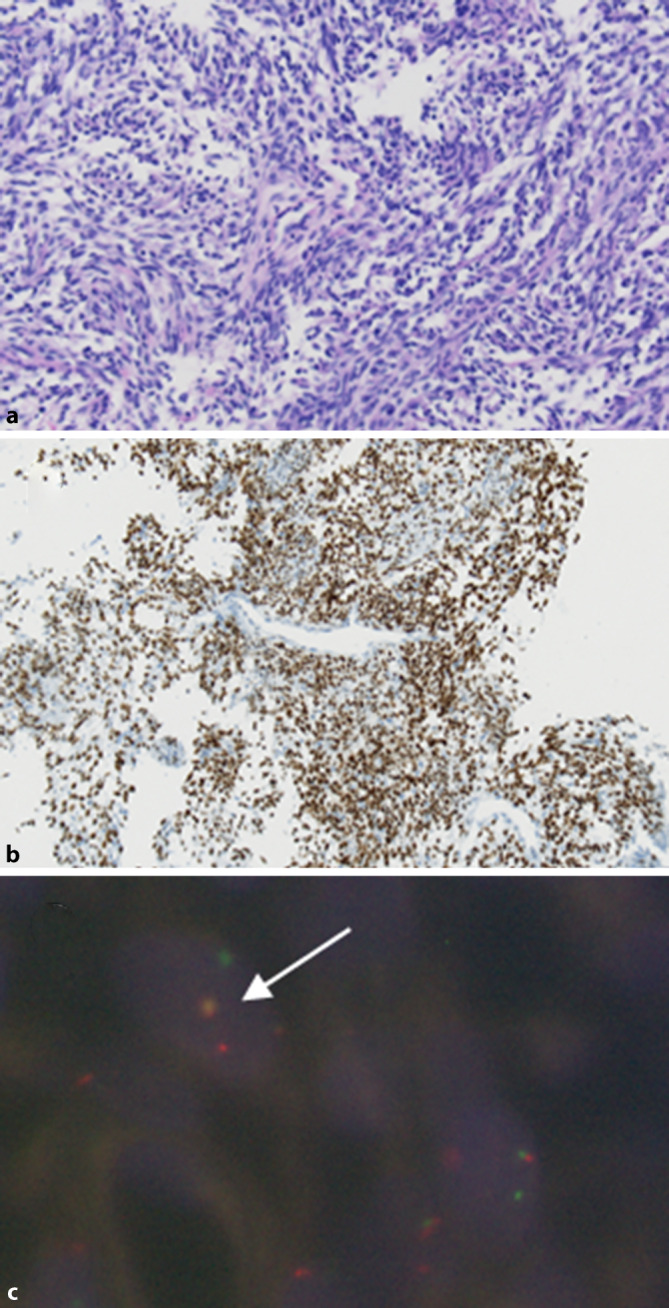

Die histochemischen Ergebnisse zeigt Abb. 1: monophasisch fibrös wachsendes Synovialsarkom des Magenkorpus (Grad 2 nach FNCLCC [Fédération Nationale des Centres de Lutte Contre Le Cancer]) mit nachgewiesener Translokation mit Beteiligung des *SS18*/*SYT*-Genlokus.

**Figure 2:**
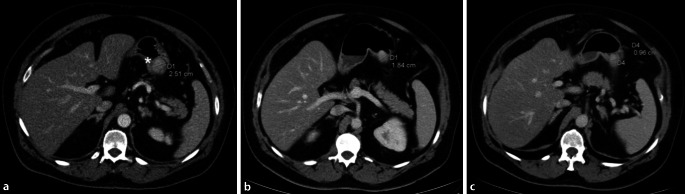

Es erfolgten drei CT-Aufnahmen. In der initialen Bildgebung (vor Induktion der Chemotherapie) stellte sich ein ulzerierender Tumor der Magenkorpushinterwand großkurvaturseitig dar (Abb. 2a). Dieser war im Verlauf (Abb. 2b) und nach beendeter Chemotherapie (Abb. 2c) größenregredient.

## Therapie und Verlauf

**Figure 3:**
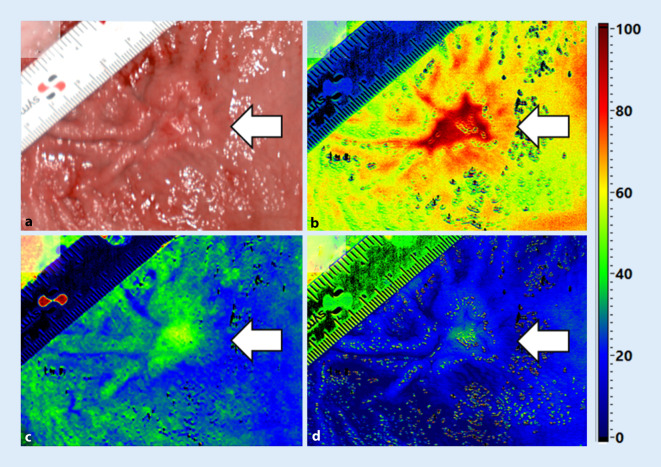

Nach Diskussion des Befunds im Sarkomboard erfolgte die neoadjuvante Therapie mit 4 Zyklen Hochdosis-Ifosfamid (2500 mg/m^2^ d1–d5) und -Epirubicin (45 mg/m^2^ d2–d4). Drei Wochen nach Abschluss der Chemotherapie und Restaging mit Nachweis eines Tumorregresses wurde die onkologische Gastrektomie mit systematischer D2-Lymphadenektomie, Cholezystektomie und Rekonstruktion nach Roux‑Y durchgeführt. Abb. 3 zeigt physiologische Gewebeparameter des aufgeschnittenen Präparats, die mittels intraoperativem Hyperspektral-Imaging (HSI; TIVITA®-Tissue, Diaspective Vision GmbH, Am Salzhaff, Deutschland) gemessen wurden.

Histologisch konnte im Operationsresektat eine komplette Regression des Tumors nachgewiesen werden. In Zusammenschau mit dem Vorbefund ergab sich folgende Einteilung nach der TNM-Klassifikation für Weichteiltumoren der Eingeweide des Thorax und Abdomens (TNM-Klassifikation 2017): ypT1, ypN0 (0/23 LK), ypL0, ypV0, yPn0, ycM0, ypR0, G2 nach FNCLCC. Die histologische Beurteilung ergab eine Infiltration in die Submukosa mit Infiltration bis in die Lamina muscularis mucosae sowie in die Muscularis propria mit unscharfer Begrenzung. Der postoperative Verlauf war komplikationslos und der Patient konnte am 8. postoperativen Tag entlassen werden. Das interdisziplinäre Sarkomboard empfahl eine postoperative Nachsorge mit CT-Kontrolle 6 Monate nach der Operation. Aktuell (8 Monate nach der Operation) befindet sich der Patient in klinisch gutem Zustand.

## Definition

Synovialsarkome sind aggressive Weichteiltumoren und machen ca. 7 % der Sarkome beim Menschen aus. Wenngleich sie am häufigsten in Nachbarschaft der großen Gelenke in den Extremitäten bei jungen Erwachsenen entstehen, können sie prinzipiell in jedem Teil des Körpers auftreten, inklusive im Magen-Darm-Trakt. Genetisch sind die Synovialsarkome durch eine reziproke chromosomale Translokation zwischen dem X‑Chromosom und Chromosom 18 t(X; 18) (p11.2; q11.2) charakterisiert, welche zu einer *SS18*-*SSX*-Genfusion führt [1].

*SS18* (GenBank Zugangs-Nr. NC_000018) wird normalerweise breit in menschlichen Geweben exprimiert, während die *SSX*-Expression auf normale Hoden und Schilddrüse limitiert ist und einige Varianten in menschlichen Tumoren gefunden wurden. Die *SSX*-Familie umfasst neun hochhomologe Synovialsarkom-X-Gene (*SSX*1–9; [4]). Dennoch machen *SSX*1 oder *SSX*2 (GenBank Zugangs-Nr. [*SSX*1] oder NC_000023) > 90 % der t (X; 18) -Translokationen aus; *SSX*4 ist in wenigen Fällen beteiligt. Die „breakpoints“ werden häufig innerhalb des Introns 10 des *SS18*-Gens und des Introns 5 des *SSX*-Gens gefunden. Die Detektion des *SS18*-*SSX* Fusiongens bzw. die Dokumentation eines Bruchs in *SS18* bei konsistentem histologischem und immunhistochemischem Profil ist beweisend für die Diagnose eines Synovialsarkoms.

## Diskussion

Obschon aktuell weltweit ca. 40 Fälle primärer Synovialsarkome des Magens beschrieben worden sind, ist die prätherapeutische Diagnose schwierig [6]. Die Diagnostik mit der RT-PCR („reverse transcription-polymerase chain reaction“) und die direkte Sequenzierung von Gewebebiopsien des Magens in Verbindung mit einer histologisch aussagekräftigen Biopsie sind möglich [3]. Zudem konnte der Nachweis des o. g. Fusiongens *SS18*-*SSX* als hochspezifischer Marker in präoperativer Plasma-cf(„circulating free“)DNA erbracht werden („liquid biopsy“). Somit gilt die Methode der *SS18*-*SSX*-Fusionssequenz in der zirkulierenden cfDNA als potenzielles Monitoringinstrument, da diese auch bei sehr kleinen Tumoren (ca. 2 cm im Durchmesser) beschrieben wurde [7].

Differenzialdiagnosen der malignen mesenchymalen Tumoren des Magens sind unter anderem gastrointestinale Stromatumoren (GIST), Leiomyosarkome, solitäre fibröse Tumoren und maligne periphere Nervenschafttumoren (MPNST; [2]).

**Diagnose:** Synovialsarkom des Magens

Das notwendige Ausmaß der chirurgischen Resektion ist Gegenstand kontinuierlicher Diskussionen: Neben Wedge-Resektionen sowie Magenteilresektionen und onkologischen Gastrektomien werden auch unterschiedliche Angaben zum Umfang der Lymphknotendissektion in der Literatur gemacht [6]. Aufgrund des relativ jungen Alters unseres Patienten sowie seines guten Allgemeinzustands entschlossen wir uns zur onkologisch radikalen Variante und damit zu einer offenen Resektion mit systematischer D2-Lymphknotendissektion. Nach diesem Vorgehen sind Rezidive selten beschrieben.

Das Hyperspektral-Imaging zeigte deutlich erhöhte Werte des Gewebewasserindex und der Gewebesauerstoffsättigung im Bereich des regressiven Tumors im Vergleich zum umliegenden Gewebe. Diese Beobachtung bestätigt vorherige Analysen an Resektaten anderer Tumorentitäten nach neoadjuvanter Therapie [5].

## Fazit für die Praxis

- Unspezifische epigastrische Beschwerden sollten auch bei jungen Patienten ernst genommen und bei Beschwerdepersistenz einer diagnostischen Abklärung zugeführt werden.
- Auch wenn Adenokarzinome, Lymphome und gastrointestinale Stromatumoren die häufigsten Tumorentitäten des Magens darstellen, existieren seltene Entitäten.
- Differenzialdiagnostisch müssen Synovialsarkome von anderen mesenchymalen Tumoren, wie u. a. gastrointestinalen Stromatumoren, solitären fibrösen Tumoren oder malignen peripheren Nervenscheidentumoren, durch histologische, immunhistochemische und molekulare Analysen abgegrenzt werden.
- Synovialsarkome sollten in spezialisierten Sarkomzentren multimodal behandelt werden. Der Therapiealgorithmus sollte in einem auf Sarkome spezialisierten interdisziplinären Tumorboard diskutiert und beschlossen werden.
- Nach neoadjuvanter Therapie können Synovialsarkome des Magens analog zu anderen Weichteiltumoren kurativ reseziert werden.
- Die Nachsorge sollte standardisiert anhand der gängigen Leitlinien erfolgen.
